# Notes from London, England

**Published:** 1887-04

**Authors:** Wm. B. Meany

**Affiliations:** London, England


### FOREIGN CORRESPONDENCE.

### NOTES FROM A LONDON SOCIETY.

LONDON, England, February 21st, 1887.

*To the Editor of* THE CHICAGO MEDICAL JOURNAL AND EXAMINER:

DEAR SIR:—I send you some notes (abstracts) taken at a meeting of the Royal Medical and Chirurgical Society.

W. E. Cant, F.R.C.S., sent a paper (communicated by Mr. Holmes), on clinical observations on

"INDURATION" IN PRIMARY LESION OF SYPHILIS IN WOMEN.

The following results are founded on 3,300 cases examined in the Royal Albert Hospital, Devonport, during a period of six and a half years.

In contradistinction to the generally accepted fact, that in men induration is as a rule well marked, it is so in women far less generally. The induration which is described as being circumscribed, limited to the base of the sore, and clearly marked off from the surrounding tissue in men, in women is, about as often as not, diffused a considerable distance into the tissues around, often very widely, not sharply defined from the surrounding tissues, nor circumscribed in the base of the sore itself. The most typical form of primary sore with its induration resembling a disc of cartilage set in surrounding soft structure, described as being frequent in men, is seldom met with in women. Every variety as to the degree of development of the induration is met with in women; but there is a large proportion in which it is very slight—about one-third.

In some of these slight cases it is impossible to affirm that there is any thickening at all present, and there is a small number in which none exists throughout the whole course of the sore.

Induration is present in the early stage of the sore in a small proportion of cases. The time of its occurrence in the course of the sore varies greatly; it may occur at almost any period of its course. It becomes most marked and developed toward the middle and latter part in many cases.

Tables of cases of primary sores, grouped according to their clinical character, and showing—when first apparent—characters at earliest period, etc., and also of five cases of infecting primary sore in which there was no induration, are appended to the paper.

Dr. R. G. Hebb's paper is entitled,

"A CASE OF ACTINOMYCOSIS HOMINIS."

W. H., æt. 11, was admitted into the Westminster Hospital on March 18th, 1886. He had suffered for a month previously from pains in the limbs, diarrhœa and "feverishness." On admission the most prominent symptoms were those of lung consolidation and pleural effusion, for which he was tapped twice. Soon after pyæmic symptoms developed, and the patient died June, 2d, 1886. The post-mortem examination disclosed abscesses of the brain and meningitis, pneumonia, excavation of the lungs, and suppurative pleuritis.

A large vegetation was found on the wall of the right auricle of the heart, and in the liver an abscess and many areas of caseation.

On microscopical examination of the liver numerous pigmented cells were found, many of which formed the center for the departure of radiating filaments. The conjunction of these two factors gives rise to the appearances of a definite organism, for which the name of Actinomyces was adopted. In the brain and lungs collections of cocci only were found.

A colloquial discussion followed the reading of these papers. The majority of the Fellows were of the opinion, regarding the first paper, that the induration around the sore would be found to exist whether in the male or female, when occupying the same class of tissues.

Regarding the second paper (Actinomycosis Hominis), it was generally concurred in by the Fellows that the history and post-mortem appearances of the case were useful in aid to diagnosis:

1st. Pneumonia existing.

2d. Wasting and hectic fever, swelling and tenderness of the region of the liver, and no fluid by aspiration.

Mention was made of three cases of this infection starting in the lung, and following the same course. One case of Mr. Israel; one case of Mr. Skillett; one case of Dr. Hebb.

Attention was called to total duration of the disease of three and a half months, beginning from ill health.

Specimens of actinomyces under microscopes (1-12 in oil) were prepared for examination.

The hour for adjournment having been announced, the proceedings were brought to a close.

WM. B. MEANY, M. D.

